# How public health nurses’ deal with sexting among young people: a qualitative inquiry using the critical incident technique

**DOI:** 10.1186/s12889-018-5642-z

**Published:** 2018-06-13

**Authors:** Maria Clark, Alison Lewis, Sally Bradshaw, Caroline Bradbury-Jones

**Affiliations:** 1 Institute of Clinical Sciences, College of Medical and Dental Sciences, University of Birmingham, Edgbaston, Birmingham, B15 2TT UK; 2 School of Nursing, University of Worcester, St John’s Campus, Henwick Grove, Worcester, WR2 6AJ UK; 3 Institute of Applied Health Research, College of Medical and Dental Sciences, University of Birmingham, Edgbaston, Birmingham, B15 2TT UK

**Keywords:** Sexting, Young people, Child sexual exploitation, Public health nursing, Health visitors, School nurses, Digital safety, Technology, Risk, Vulnerability

## Abstract

**Background:**

Globally, the potentially harmful effects of using cell phone technology for ‘sexting’ among young people, is a public health concern. The background literature indicates that sexting might have adverse psychosocial consequences for some young people who share partially nude images (‘selfies’). Public health nurses (PHNs) could offer guidance to children and young people on digital safety, yet little is known about their role in this regard. This study explored PHNs’ knowledge and confidence in addressing the issue among young people.

**Method:**

A qualitative study was undertaken using the Critical Incident Technique. The study took place in 2016. Eighteen semi-structured interviews were conducted with PHNs in a region of England. Data were analysed through thematic analysis, and managed through the use of NViVo 11 software. From the entire data set, thirteen critical incidents were identified of which nine were deemed relevant for reporting in this paper.

**Results:**

PHNs regarded sexting as a contemporary ‘normalised’ practice that takes place in what young people consider to be trusting relationships. PHNs’ knowledge was informed by media reports that supported their beliefs about young peoples’ vulnerability to risk-taking sexual behaviour. They were not confident about discussing sexting with young people, even though some PHNs had done so in light of concerns about potential child sexual exploitation.

**Conclusion:**

PHNs have a role to play in advising young people on digital safety, but findings of the study show that their role is not fully realised. They have some knowledge of sexting as a possible signifier of abusive behaviour. However, they are not always confident in dealing with the issue. Improving PHNs ability to promote digital safety through better understanding of technology use among young people is good safeguarding practice. This may, in turn, better define this important nursing contribution to public health.

## Background

Globally, protecting children and young people from potentially ‘harmful sexual behaviour’ (HSB) is a public health concern [1–11]. The international literature exploring the impact of sexting and social media on adolescent health has grown in recent years [4–10]. Literature from UK, Europe and USA indicate that sexting is a popular activity that might have profound consequences for some young people. The UK government and reputable independent organisations have recently produced guidance on the safe use of social media, suggesting the potential benefits and risks for adolescent health and wellbeing [7–10].

Sexting has arisen during the twenty-first century as the distribution of self-produced cell phone images has become a regular activity which can include images of a sexualised nature. Doring’s comprehensive review included 50 studies from Europe and USA and found that whilst the emergence of sexting has been viewed as mainly a youth phenomenon, sexting is also commonplace amongst adults. However, adolescents are getting steadily more involved as they grow in age [5]. The idea that sexting might be harmful is controversial. In the UK, the Sexual Offences Act (2003) may not have anticipated the criminalisation of adolescents due to sexting. Recently, the national press debated whether it was fair, suggesting that ‘making mistakes’ is part of growing up [12]. Lenhart’s survey in the USA found that legislatures in some states were considering downgrading the charges for creating or trading sexually suggestive images of minors by text from felonies to misdemeanours [13]. These authors noted the prevalence of sexting amongst the 12–17 age range was 4% of all cell-owning teens with little difference in gender. Older teens (17 year olds) most commonly reported sending a sexually explicit image with text, and one in six claimed to have received a sexually explicit image from someone they know. Ringrose and Dobson showed that social media ‘tagging’ of images is important to adolescent peer recognition. However, in cases where girls’ digital interactions were inappropriately shared or misrepresented, social tagging was perceived by girls as a disempowering public transgression [14].

Houck and colleagues argued that flirtatious phone messages in early adolescence may be a co-occurring sexual harm; they say the use of cell phone technology for ‘sexting’, or ‘sharing nudes’ shows a weak correlation with other adolescent sexual activities, including unprotected sex [6]. West and colleagues undertook a large survey of 949 high school aged adolescents in Peru, and claimed significant correlates for girls’ sexting; risk factors included having been cyberbullied and parental factors. For boys, hyper-texting, fighting, and parental rules about sexting were significant [15]. In a survey conducted by Benotsch, with 763 young people in the USA, sexting is robustly associated with high-risk sexual behaviour and/or using substances [16]. Gomez and Ayala’s review highlighted possible psychological aspects associated with harmful forms of sexting. They suggested personality traits such as more extreme impulsiveness, were displayed by some sexting teenagers involved in criminality [17]. Dake et al.’s survey of American adolescents showed statistically significant correlations between sexting and sexual behaviours, substance use behaviours, emotional health behaviours, and time spent texting. These authors recommended that sexting should be addressed by parents, teachers, and mental health professionals who interact with adolescents [18].

Others, such as Ferguson, and Gorden-Messer et al., refute this viewpoint [19, 20]; Ferguson found sexting was un-related to most sexual risk behaviours in a survey of young female Hispanic students in the USA. 20% of participants reported engaging in sexting, associated with sex without contraception, perceived pleasure of sexual activity, and 'histrionic' personality traits, which did not correlate with increased number of sexual partners or unprotected sex with new partners. Gorden-Messer et al. compared sexting among teenagers who described themselves as sexually active and those who did not and found that sexting was not related to sexual risk behaviour or psychological harm in either group.

Thus, while the evidence is somewhat equivocal, most studies have found that sexting ‘auto-correlates’ with other sexual behaviours such as flirting, dating, and having sex/sexual intercourse. Sexting behaviours relate to other social factors, such as whether teenagers are in sexual relationships and how or whether they use social media to engage with their peers. In a recent study of the impact of social media use and adolescent mental health, Berryman et al. suggested that social media use among young people was not predictive of impaired mental health functioning, although they found the *quality* rather than the quantity of use is relevant; ‘it’s not *what* you use that counts as much as *how* you use it’ [21].

Internationally, public health nurses (PHNs) (including family nurses, health visitors and school nurses) are uniquely placed to identify adolescent health needs. Through home visiting and school support nurses can undertake risk assessments and offer guidance to children and young people using social media and electronic devices [11, 22–24]. However, the scope and impact of the PHN role in this regard is unknown. PHN (school nurses) may have to initiate a response when a sexting episode is reported as an issue [11, 22, 23]. There is an emerging body of empirical evidence and policy development in relation to sexting generally, but to date, the role of PHNs in dealing with this remains un-investigated.

## Methods

### Aims & research questions

With this contextual backdrop in mind, the aim of the study was to investigate PHNs’ knowledge and confidence in dealing with the issue of sexting among young people.

The research questions were:

1. What are PHNs’ understandings about sexting and its impact on health and wellbeing of young people?
2. How confident are PHNs in discussing the issue of sexting with young people?
3. How do PHNs describe their role in promoting the health and wellbeing of young people in relation to sexting?
4. What are the practice, education and political factors that might influence PHNs’ ability to deal confidently and effectively with the issue of sexting among young people?

This was a qualitative study into which we integrated the Critical Incident Technique (CIT). The hallmark of a CIT study is a focus on ‘real’ events, rather than abstract concepts [25–27]. It involves asking respondents to recount *actual* incidents. We were interested in how PHNs discuss sexting with young people and their perceptions of preparedness to deal with the issue. CIT allowed us to access as closely as possible the realities of PHN practice expressed through example, rather than hypothetical statements. As far as we are aware, we are the first to report qualitative research with professionals on this issue.

### Ethics

Ethical approval was obtained via the local NHS Foundation Trust - Integrated Research Application Service (IRAS 197125). Participants were invited to join the study and were given written information leaflets prior to taking part. Informed, written consent was gained from all participants.

### Recruitment

PHNs (*n* = 18) were recruited from one locality in England using purposive sampling. Purposive sampling is commonly used in qualitative research as a means of recruiting participants who share experience of a certain phenomenon; in this case, dealing with sexting among young people. We had aimed for a sample of 15 because this was considered sufficient to allow for meaningful insights to be gained, but manageable enough regarding the potential to generate significant volumes of qualitative data. Sample size in qualitative research is contentious, particularly when considering the issue of data saturation [26, 27]. A sample size of 12 is considered to be sufficient in most studies with a homogeneous sample to achieve data saturation [28–30]. However, we were not convinced that data saturation had occurred at that point. Therefore, we continued to recruit until no further new insights were generated.

To be included, the PHNs nurses needed to be practicing currently within the field of public health nursing (as a family nurse, health visitor, or school nurse) and working with children and young people within the selected research site in England. In the first instance, nursing clinical leads were approached via written introduction to the project and asked to discuss the request for participation at a team meeting. Any nurse who was interested in participating was then contacted by the lead researcher and further information sent. Consent was obtained at interview. The nurses who participated were based in the same locality and equal numbers of school nurses, health visitors and family nurses were interviewed.

### Data generation and management

Data were generated through semi-structured, 1:1 interviews undertaken between May and August 2016. To ensure consistency aligned to real world practice all interviews were conducted by AL, a practising PHN researcher at the time. A simple interview schedule was designed using CIT principles as a guide (Table 1) drawing from Bradbury-Jones and Tranter’s guidance [26, 27]. In line with the CIT methodology, participants were asked to recall ‘real’ incidents where they had addressed the issue of sexting with a young person. In order to capture the fullest incidents possible, the interview guide encouraged participants to provide an account that included an antecedent, the situation/context and consequence. All interviews were audio recorded and subsequently transcribed verbatim. To protect participants’ anonymity, they were assigned a code from Participant 1 to 18. Data were analysed through peer-reviewed thematic analysis and managed with NViVo 11 software [31]. Nine critical incidents were deemed relevant for reporting in this paper.

**Table 1:** Interview guide

Are you able to give me an example of engaging with a young person where you consider they may have shared a sexualized image?	
Follow up questions	
What happened? (Context)	
What led into this situation? (Antecedent)	
What was your reaction?	
How did you feel about the situation now?	
What was the outcome? (Consequence)	

## Results

A summary table of themes identified from the CIT guide is provided in Table 2.

**Table 2:** Summary themes derived from CIT

Participant #	Antecedents	Context	Consequences
3	Young people not realising the consequences Young people thinking sexting is ‘a bit of a laugh’ Lack of knowledge about sexting – only heard of it ‘in the news’. Never talked about sexting in role.	Public health nurse lacked knowledge Critical incident linked to media reports Media report of sexting. PHN shocked.	Perceived need to avoid normalisation Perceived need for education Public health nurse more aware as a result of taking part in the study PHN has thought about sexting more as a result.
5	Thinking that the relationship is a trusting one Being let down Ongoing relationship with client. Already talking about trust.	Young girl being able to discuss matters with the public health nurse Client thought she was in a trusting relationship. Photos were shared. One of PHNs least complicated clients. PHN took no further action as client did not want to take it further but left it open to talk about again in the future.	Being informed in how to respond, rather than ‘blagging’ Educating on power and control within relationships PHN would not do anything differently now but would like more information on how to respond, rather than ‘blagging’
6	Genuine relationship ‘gone wrong’ Young people not realising the consequences Experience of sexting as part of child protection cases where involving 15 and 16 year olds.	Sexting as a feature of contemporary culture 15 year old has ended up on a child protection plan	Practice experience of legal consequence i.e. criminality – Sexting can ruin lives PHN has reflected on need for messages to be clear and direct, informing young people that the legal consequences can ‘ruin your life’
9	Beginning the conversational process in the first place PHN has experienced sexting in professional role a number of times.	It’s a normal practice for some young people Used opportunity to discuss risks of sexting and how to stay safe. Client thinks differently about how she uses pictures as a result. Learning also shared with friends.	Don’t have images in the first place Sexting can be dangerous PHN demonstrates confidence in using sexting issues as an opportunity for health promotion and harm reduction.
14	Girl has learning difficulties Client known to PHN as known child protection case due to neglect. Probable learning disabilities. Parents not able to protect her on line.	Family on social services radar Paradox of children already being at risk being less of a concern Client sharing pictures of self in underwear over Facebook. Family ‘on the radar’ and known to school therefore this was identified and dealt with.	What happens when a child is not ‘on the radar’? Educating on what is appropriate to share PHN involved teaching her how to protect herself on line. PHN has ongoing worries for children ‘not on the radar’.
17	Girl has learning difficulties (risk factor//vulnerability) One girl disclosed to a (teacher) that she had shared explicit images of herself. Second girl with learning disabilities involved in on-line grooming involving sexting.	Public health nurse is confident in discussing sexual health PHN ‘surprised’ despite being used to young people discussing sex ‘vividly’ with her. Both cases were dealt with as child protection cases (my wording might not be very good here)	The young girl hadn’t ‘learnt’ Vulnerability Absconding Second girl ‘disappeared’ and met up with a man she met on line when she was 17.
7	Grooming retrospectively identified Blackmail Girl has learning disability PHN feels she doesn’t know a lot about sexting and hasn’t had a lot of experience. 12/13 year old girl blackmailed into sending explicit photograph of herself to him via Facebook. Led to her then being blackmailed in to having sexual intercourse with a 15/16 year old.	Friend of girl ‘took it into her own hands’ and involved teachers and parents. PHN identifies it as grooming but also states that the girl ‘allowed’ herself to be blackmailed.	Girl still didn’t seem to be worried about the situation. PHN expresses feeling helpless, and cross and that there ‘must be something we can do to protect them from this and I don’t know what that is’.
10	Grooming retrospectively identified PHN involved in case through safeguarding meeting that a girl with identified as involved in CSE (child sexual exploitation) was sending images and being videoed.	Debates about consent and abuse Legal issues - Police don’t know whether prosecution possible as in she is giving implied consent in some videos and not others. PHN identifies child as victim of grooming.	What happens when a relationship breaks down? PHN worked with girl to explore her understanding of sexting and safety, and to think about what may happen if a relationship breaks down.
13	PHN identifies a 14 / 15 year old girl with a learning disability who was exploited by a paedophile who was grooming her, and she sent explicit images. PHN states that the girl has a high sex drive and uses telephone sex lines. Grooming suspected Predatory Paedophile (retrospectively identified) Girl has learning disability Girl has high sex drive Girl craves attention	Girl was wrong No discussion of actual actions in relation to this case.	Long term mental health problems Pregnancy Violent relationships PHN reflects on possible long term health consequences: depression, anxiety, suicidal thoughts, and potential for pregnancy and violent relationships.

Within the entire data set we identified 13 critical incidents. Of these, nine had a direct or indirect relationship with PHN practice, while four related to personal, rather than professional experiences. To retain the integrity of data, we have presented the nine critical incidents in full in this section. For the sake of readability, critical incidents have been subject to minor editing and ‘cleaned’ for grammatical clarity. In addition seven participants gave valuable insights into the issue of sexting as a hypothetical concern (reported elsewhere) but they were unable to relate the issue directly to practice by way of example. As an important finding in its own right, this reveals a gap in many PHNs’ role in dealing with the issue.

### Normalisation and trust

The first four incidents capture how a ‘normalisation’ of sexting takes place in what young people considered trusting relationships (Participants 3, 5, 6 & 9). PHNs also discuss the impact of national media reports on their knowledge acquisition in this regard.

#### Participant 3

This is how little I know! But I do remember there was a case [published in the press] and he was charged with the offence… It’s probably something I’ve only heard on the news; a few cases that have made my ears prick up. When you first mentioned it, I don’t think I’d heard of it and I’m probably more aware of it because you’ve been doing a study on it. As a family nurse I think I should be. I don’t think I’ve ever talked about sexting.

*Interviewer:* If you think back to the case that you mentioned in the press, what was your reaction when you first heard about it?

It was quite shocking to be honest… I thought you could see how young people could get into this without really realising the extent of how it could end up and not understanding it really. That’s where the education comes in from the teachers and professionals because [to young people] it’s all a bit of a laugh… We don’t want to normalise it do we? It’s interesting. We were talking about sexting earlier with colleagues and some had never heard of it at all. My knowledge feels very scant, but I think, only because probably you’re doing this study and I’ve heard it also mentioned a couple of times on the news, that it made me really think about it, so it’s been a good, positive outcome really.

In contrast to Participant 3, who felt that young people might trivialise the issue of sexting as a normalised feature of their intimate relationships, Participant 5 used discussions about trust and relationships to discuss sexting with one of her female clients. For her, the risks associated with sexting were around being let down within a relationship, and exploring power and control within intimate relationships.

#### Participant 5

[One young girl] shared with me when we were talking about trust, I think, or relationship stuff, she was able to say, ‘well actually I did have a partner who I thought I could trust and… he shared these pictures with his friends and what have you’. She didn’t do anything about it so it never went anywhere. She’s never done anything about it. I guess I empathised with her in the first instance and then said, ‘That’s not right’, and tried to get her to think about if it was an equitable relationship or whether there was power and control in there. But she didn’t want to do anything formally about it… but she was talking about it, ‘Yes it was really rubbish… why would he do that if he said he loved me?’ … She’s one of my least complicated clients but she just trusted this chap who let her down big time.

*Interviewer*: When you reflect on it now, would you have done anything differently when she told you?

I don’t think so… I left it with her, left it open that if she wanted to talk about it again we could explore it further if she felt the need. But she hasn’t done. No I don’t think I would do anything different other than maybe knowing what to do: To be able to say: ‘Actually if X happens, you go to Y’, or those sorts of things because really, when talking about that bit, I was just blagging [making it up].

Similarly, Participant 6 also discussed the risks of sexting as being about a genuine relationship going wrong, with young people not foreseeing the potential outcomes when this occurs. This participant suggested sexting is a normalised ‘culture’ among young people and educational interventions need to deliver strong messages that convey how sexting can ruin lives.

#### Participant 6

We’ve had quite a lot of child protection cases going on where young people who have been 16 years old have texted something to somebody who was 15 and this has led them to be on a child protection plan… when actually, it was just a genuine relationship that has gone wrong with sending images of each other and parents have seen those images... I think that because they were so young it was like, ‘Oh, really? You complete idiots! Fancy doing that to yourselves’. But it’s their culture isn’t it? In this day and age this is how they feel they have to communicate. I haven’t had any real conversations with him [the boy involved] because he’s been dealing with other agencies…They [young people] don’t understand the consequences even though you talk about somebody being of a legal age and somebody not, it doesn’t come to the front of their minds, [they think] ‘that’s my girlfriend, we are in the same year at school, what can be so different about us?’ You can’t do a lesson plan for an hour because they’ll switch off after 10 min. We need something that they’ll pick up on at the beginning that says: ‘It could ruin your life if you do x, y and z’.

Participant 6 had some experiences of child protection involving sexting that had led her to think about best practice. She made some judgments about how harms could have been avoided although she had not spoken with those involved. She suggested a need for tailored educational interventions, although she had not yet followed through. On the other hand, participant 9 had undertaken direct conversations with the young people involved:

#### Participant 9

It’s not just been one family I’ve worked with, it’s happened a few times: One of the first families that I came across… it was an opportunity to really talk to the client [young girl] about the importance of not having these images to start with and how dangerous they can be. She was able to reflect on the impact it already had on her life and her circle of friends and her family, all from enabling pictures to be taken and that kind of involved, you know sexting. It was an opportunity to get the client’s understanding of what it was and how severe things can go and she had quite close friends and they all started to kind of realise how important it is not to allow partners or whatever to have these images of them to start with, so that they’re not in that situation. But other people may well think this is still quite normal. It’s definitely - she [young girl] certainly told me at the time that it’s really made her think very differently to how she uses pictures.

This report of a conversation in practice demonstrates shared learning that led to a change in a young person’s perspective of harms involved. A further important theme links to young girls described here as being particularly 'vulnerable' to child sexual exploitation. This finding was perceived by PHNs to be due to the girls’ having additional learning difficulties (Participants 14 & 17) or learning disability (Participants 7 & 13).

### Vulnerability and child sexual exploitation

Child sexual exploitation (CSE) is associated here with sexting behaviour. Two PHNs identified child protection needs which suggested a normalisation of high risk sexual behaviour among vulnerable, young, learning disabled girls. This was expressed to the point of PHNs being ‘unsurprised’ by the girls’ graphic sexual detail of encounters with men who they perceive as boyfriends. Participant 14 identified her educative role in supporting a vulnerable 13 year-old girl to stop sharing intimate selfies, while participant 17 describes the feeling of helplessness in addressing the issue.

#### Participant 14

There was a family where the children would always come back into Child Protection [the attention of social services] through neglect. The daughter in that family, she probably had borderline learning difficulties. My involvement with her began when it came to light that she was using Facebook and sharing everything and she had requests to show pictures of herself in her underwear… and she did. She was 13. Thankfully it came to light at school and school involved everybody appropriately. I became involved around educating her about how to protect herself; how to help her with understanding about relationships and what’s appropriate to share and what isn’t appropriate to share.

*Interviewer*: When you first heard about that what was your initial reaction?

Horror really! I thought she’s really at risk… I was really frightened for this girl because I knew the parents didn’t have the wherewithal [to educate and protect her]. I was very worried about her safety really… In some ways this girl was in a good position because her family were definitely on the radar with school because her parents were well-known to school…. My worry would be for a child whose family aren’t on the radar… So my worry would be for another child.

#### Participant 17

A young person disclosed to one of the members of staff that she had some explicit images of herself and she’d sent them to somebody. And an image had obviously been shared by lots of people so we were involved at that stage with kind of child protection issues… I was surprised a little bit but.... You get used to what they’re telling you… you get to the stage where perhaps nothing surprises you quite so much. We had another young girl. She’s got learning disabilities and she was going on the internet and she was meeting… well, she thought they were her boyfriends, she thought she was in a relationship… It’s very difficult, you know. We’re supporting her and trying to advise her and also school were aware of what she was doing and they were trying to support her about not doing it. But she thought she’d met the love of her life and it was like, you know, ‘this is my boyfriend’. But she hadn’t even seen him face-to-face… But she had got some learning needs so we were involved with her on the edge of the child protection arena you know? [Unfortunately, when she was 17] she disappeared and they didn’t know where she’d gone and she did actually go to meet one of these men down in London. So… she hasn’t actually learnt and she’s obviously a very vulnerable young lady.

The predatory nature of sexting and the grooming that can take place was evident in the incidents reported by the following three participants (Participants 7, 10 & 13):

#### Participant 7

I’m aware that I don’t know an awful lot about it and I haven’t had an awful lot to do with it, but I have had a couple of incidents with indecent images of girls being, for want of a better word, blackmailed. One incident I’m particularly thinking about it was like a young girl, sort of year 8 [12–13 year-old] and she had a year 10 boyfriend [14–15 year old], who wasn’t a particularly nice guy and who said to her, if I remember this to be correct that, unless she took a photograph of her genitals and sent them to him on Facebook, he was going to tell everyone that she had sex with him. So of course she did that. But he felt the need to pass it on to other people anyway. One particular young man who was year 11 [15–16 year-old] then said to her, ‘I know where you live and unless you agree to have sex with me, I will come round to your house and tell your mum’. This girl, fortunately for her had a really good friend and her friend took it into her own hands and went to the head teacher and then of course it was taken up from there and the parents were involved. It might come across as sounding naive but you would hope that they wouldn’t be so cruel at that age wouldn’t you? At first, I thought, ‘Hold on a minute’, because I didn’t expect it in one so young. I thought they’d pick on girls who were maybe aged 14, you know? I was shocked. I was angry. It was a little bit odd because she didn’t seem too worried about it. Her friend seemed to be more worried about it than she was. I don’t know whether that was something to do with her learning abilities or not, to be honest. But I just thought how vulnerable did that make that girl or, ‘you’ve done this now’, you know, she’s allowed herself to be blackmailed... It scares me because we try and protect them but I’m thinking it’s so difficult to protect them from this sort of thing, isn’t it? If you tell some young people about these sorts of dangers it’s all, ‘Yeah, we know about that’. They all feel that they know a little bit about it. I think sometimes it’s done so insidiously, it’s grooming isn’t it? I feel that there must be something that we can do to protect them from this and I don’t know what that is, you know? It makes me cross because it makes you feel helpless doesn’t it so far as that’s concerned.

#### Participant 10

It’s something I haven’t really thought about a lot until recently. One of my caseloads involved with CSE (child sexual exploitation), made me start thinking of sexting, because at a meeting it was brought up that this young girl was actually sending photos of herself and being videoed, so I think I’ve only really become aware of it in the last few months.

*Interviewer*: How did you react, what was your reaction when you heard about what this young person was doing?

Quite shocked! The police were saying they’d seen the videos and that she obviously was giving consent and in the other videos they feel that she wasn’t, so the police said that they struggle whether they can prosecute because they don’t know where they stand and that’s really hard, because to me, that girl needs support and help. It’s grooming, isn’t it?

*Interviewer*: So how do you think you can best take it forward or support that client?

Discussing with her what she was thinking when she sent them. Was she aware that they could be passed on? The girl’s saying that she didn’t know some of them were taken, so I would try to find out what she means and explore it more and I think to me, trying to make our clients aware that they might be in a relationship, they are sending these texts out, but what happens if that relationship breaks down? Where are they going to go then?

#### Participant 13

I’ve had one [young girl] where he was getting her to send pictures of herself and he was actually a paedophile and he was grooming her… it is all exploitation… It’s a massive issue and with these girls or even boys, they’ll carry that with them for the rest of their lives, so it could start triggering depression, anxiety, suicidal thoughts, it’s a massive mental health issue…It’s awful. I was really angry because I knew the family fairly well and she’s clearly got a disability, a learning disability, so she’d been exploited… I knew that she had quite a high sex drive, so she was frequenting sex telephone lines and other things, so I was horrified by the fact that this man could happily exploit this girl to get her to send images of herself … To this day, I wouldn’t know whether she is aware that what she did was wrong… She is 14, maybe 15 years old and she’s got a very high sex drive, which is very common within this group. She was seeking out her sexual fantasies through these chat lines… These men will pick them out, they just know. They, they can see the vulnerability and they just draw them [young girls] in and he gave her a lot of attention, because she was very attention seeking. [The problem is] we don’t want them [young girls] getting pregnant, we don’t want them making relationships with these men, that creates trouble for them and they end up getting into violent relationships and that sort of thing.

In these latter critical incidents, while talking about the grooming of young girls with learning disabilities, the PHNs’ discourse of normalisation shifts to talking about sexual harm in a way that could be construed as blaming. Some of the judgments about normalisation and vulnerability relating to sexual behaviour reflected PHN beliefs and attitudes about sexting as a feature of youth culture. The focus on the predators is relatively absent or abstracted, except when young boys were involved, perhaps reflecting PHNs’ lack of knowledge about the harmful intimate relationships the girls were involved in outside of school or in their local communities. In considering best practice these incidents raise questions about perceived vulnerability to child sexual exploitation and the PHN role in identifying and responding appropriately to sexting as a signifier of harmful sexual behaviour. The incidents demonstrate occasional PHN attempts to address the issue, and suggests the limited capacity of PHNs to raise their concerns about sexual predators identified or associated with children and young people’s sexting.

## Discussion

This qualitative study draws together the distinct issues of young people’s sexting and PHN practice. In this study the PHNs suggested that sexting was an issue affecting young people in their local practice. They reflected on real world critical incidents to support their thinking about the legal and health harms identified and their limited opportunities to deal with the issue. Their professional testimonies add weight to the literature that shows how sexting behaviour is sometimes associated with criminality [12, 15] and adverse mental and sexual health [9, 10, 13–15]. It is important to be knowledgeable about harmful sexual behaviour and realistic about the level and likelihood of risk. PHNs are well placed to identify and respond to the issues young people face but need to be technologically prepared if they are to identify potential harm and promote digital safety for young people’s health.

The PHNs in this study suggested sexting has become normal/normalised. This was evident in their discussion of their knowledge of families perceived to be at risk of harmful sexual behaviour. Yet through discussing their difficulties in reporting, and in defending what appears to be consensual sexting, there was little discussion about sexual coercion, or trajectories of abuse from seemingly healthy to unhealthy sexual relationships. Some of the assumptions and judgements about youth culture and sexual behaviour could be challenged or resisted by young people themselves, especially as sexting was rarely discussed with them. However, at the time of this study the PHNs had limited knowledge or engagement with young people’s use of social media. They did not suggest they were aware of the possible health-related benefits of social media/apps or other technological resources. Technological literacy is an area that warrants attention if PHNs are to meaningfully engage with young people using social media and technology to develop sexual relationships.

Online grooming for harmful sexual activity was identified by PHNs, after the event, in retrospective realisation of predatory relationships. Some PHNs suggested that girls in particular are more likely to display underlying vulnerability to sexual abuse, usually involving sexualised or disinhibited behaviour. However, the PHNs did not always suggest the need to know more about the abusive relationships in which such behaviour was reported. Where sexual and mental health risks were identified, this was superficially described and could be construed as moralising. Risky behaviour, such as young girls sharing nudes through online relationships with much older men, was identified as ‘high sex drive’ in one case, minimised through lack of formal reporting. PHNs generally ascribed to the idea of a normalised ‘culture’ of risk-taking behaviour. Children and young people living with learning disability figure in the PHNs’ examples of risky behaviour; supporting the international evidence that learning disabled children and adults suffer compounding risks of abuse [3]. Although some PHNs appropriately identified grooming of young girls with learning disability, others resorted to inaction, only partially identifying unhealthy environments or predatory relationships in their assessment of sexting as a risk factor.

Interviewing PHNs through CIT allowed for a deep reflection on a rarely explored area of practice [32]. Bradbury-Jones and Tranter have discussed inconsistent use of the CIT methodology in nursing research [26, 27] and the relatively small sample size here could be considered a study limitation. Nonetheless, we obtained contemporary practice data from 18 PHNS in this region. The abstraction of the nine critical incidents from the full interview data could be considered a qualitative shortcoming. However, we believe the incidents demonstrate their practical worth, supporting the assertion of Yonas and colleagues regarding the value of CIT in exploring sensitive issues in real situations [28, 32]. Each incident referred to the research aim [30]; demonstrating how PHNs responded to the CIT approach to reflect on their knowledge and confidence in dealing with the issue. Thus, although locally situated, CIT could be used to undertake similar conversations with PHNs in different settings. In this sense, our small-scale qualitative research is relevant and transferable to wider contexts of practice.

At the time of the study there was relatively little practice, education or policy guidance to inform PHNs about their role in assessing adolescent sexual behaviour. This perhaps limited their capacity to reflect deeply on the sexting issues they associated with child sexual exploitation. The critical incidents helped identify how PHNs deal with real world sexting issues as possible signifiers of sexual or mental health harms for some young people. They did this through regular home visiting and linking to schools; raising awareness among children and young people of the potential harms of sharing nudes through cell-phones. PHNs have greater independence than most nurses, and their role in child protection can be likened to an emergency service [1]. The PHN role in child protection is challenged by global depletion of the workforce and lack of child protection training [1]. In this study many PHNs did not fully realise the potential risk relationship between sexting and child protection; or discuss recommended pathways of referral to specialist services [8–10].

To some extent it can be argued that all PHNs did not (or could not) realise their child protection role in preventing potential harms associated with sexting. As mentioned, this was evident in the few interviews where sexting behaviour was attributed to young people’s sex drive or culture, even when harm was identified. Thus, it is vital that PHNs are mindful of multiple signifiers of abuse, asking young people about co-occurring sexual activities, including sexting. PHNs appear to need support and training to undertake crucial conversations about technologically mediated sexual behaviour among young people.

In this study the use of CIT captured previously unreported PHN views and opened a dialogical space for practitioners to reflect upon their experiences. The findings suggested that while most PHNs do identify young people at risk of sexual harm, there is a gap in their knowledge and confidence in dealing with the issue of sexting. They are under-confident in responding to technological changes in society and it is likely that opportunities for promotion of adolescent sexual and mental health may be missed. This paper might serve as a prompt for action to support PHNs in engaging with technologically-mediated sexual health issues, promoting digital safety among young people.

## Conclusions

The study findings support existing literature, suggesting that young people’s sexting is sometimes a signifier of harmful sexual behaviour. The nine critical incidents identified, suggested the PHN role in risk assessment is enabled through regular contact with young people in their homes and in schools. However, for the PHNs in our study, understandings about sexting were informed by media accounts, anecdotes and local safeguarding issues that were retrospectively identified. For most PHNs, the research study was the only opportunity for reflection about sexting as one of a cluster of potential risk factors associated with digital technology. PHNs were under-confident about discussing the impact of technology on health and not confident at all about discussing the issue of sexting with young people, even though some had done so in light of concerns about potential child sexual exploitation.

Although PHNs have an important role to play in protecting and promoting young people’s health, the findings from the study suggest that their role is not yet fully realised. They are uniquely placed to improve safeguarding knowledge through regular home visiting and health based links to young people in community clinics and in schools. Their contact with children, families and young people, however limited, is a potential protective factor. However, they are not well prepared for identifying and responding to the technological advances that signify children and young people’s lived experiences in the digital age. Critical incident technique demonstrated how PHNs *can* respond to sexting among young people and alongside the literature it sheds new light on PHN understandings of perceived harms. Improving PHNs’ ability to identify potential harm and promote digital safety through understanding technology use among young people is good safeguarding practice. This may, in turn, better define this important nursing contribution to public health protection.

## Abbreviations

NHS: National Health Service
NIHR CLAHRC: National Institute for Health Research Collaboration for Leadership in Applied Health Research and Care
PHN: Public Health Nursing
USA: United States of America
